# Biomechanical alterations during gait following partial ACL injury and the effectiveness of anatomical reconstruction: an *in–vitro* robotic investigation

**DOI:** 10.3389/fbioe.2025.1546180

**Published:** 2025-03-26

**Authors:** Jinpeng Lin, Rongshan Cheng, Yuan Yan, Xiaolong Zeng, Wenhan Huang, Chunlin Deng, Tsung-Yuan Tsai, Shaobai Wang, Yu Zhang

**Affiliations:** ^1^ School of Materials Science and Engineering (National Engineering Research Center for Tissue Restoration and Reconstruction), South China University of Technology, Guangzhou, China; ^2^ Department of Orthopaedics, Guangdong Provincial People’s Hospital (Guangdong Academy of Medical Sciences), Southern Medical University, Guangzhou, China; ^3^ Guangdong Engineering Technology Research Center of Functional Repair of, Bone Defects and Biomaterials, Guangzhou, China; ^4^ School of Biomedical Engineering and Med‐X Research Institute, Shanghai Jiao Tong University, Jinan, China; ^5^ Engineering Research Center for Digital Medicine of the Ministry of Education, Shanghai, China; ^6^ School of Medicine South China, University of Technology, Guangzhou, Guangdong, China; ^7^ Department of Orthopaedics, Guangdong Provincial Hospital of Chinese Medicine, The Second Affiliated Hospital of Guangzhou University of Chinese Medicine, Guangzhou, China; ^8^ Postdoctoral Workstation, Guangdong Provincial Hospital of Chinese Medicine, Guangzhou, China; ^9^ Key Laboratory of Exercise and Health Sciences of Ministry of Education, Shanghai University of Sport, Shanghai, China

**Keywords:** anterior cruciate ligament, joint contact pressure, partial injury, anterior cruciate ligament reconstruction, gait

## Abstract

**Background:**

The biomechanical alterations of the knee throughout the gait cycle following partial anterior cruciate ligament (ACL) injuries remain unclear.

**Purpose:**

This study aimed to investigate the changes in intra-articular contact mechanics during gait following partial ACL injury and to evaluate whether anatomical single-bundle ACL reconstruction (ACLR) could restore these altered mechanics.

**Methods:**

Seven fresh-frozen cadaveric knee specimens were used to evaluate tibiofemoral joint biomechanics under three ligamentous conditions: intact ACL, anteromedial bundle deficiency (AMD), and single-bundle ACLR. A 6 degree of freedom (DOF) robotic system simulated gait motion using physiological loading conditions derived from human. Biomechanical parameters, including peak contact stress, displacement of contact center of stress (CCS), and regional loading patterns, were analyzed at five key gait cycle stages. Statistical analyses were performed using repeated-measures ANOVA and paired t-tests, with significance set at p < 0.05.

**Results:**

AMD knees demonstrated a slight posterior shift in the CCS (<2 mm) during the stance phase, with significant increases in medial compartment regional loading at heel strike (4.11 ± 1.5 N, p = 0.04) and terminal stance (6.31 ± 1.35 N, p = 0.048). ACLR knees exhibited greater posterior CCS displacement in the lateral compartment at heel strike (2.73 ± 1.98 mm vs 0.21 ± 1.97 mm, p = 0.022). The sustained posterior shift in CCS will lead to abnormal loading at the posterior horn of the lateral meniscus, potentially accelerating meniscal tears or degeneration and increasing the incidence of lateral osteoarthritis. Additionally, ACLR knees exhibited significant force increases across both compartments, including the lateral compartment at terminal stance (11.91 ± 2.58 N, p = 0.027) and the medial compartment at pre-swing (11.72 ± 2.17 N, p = 0.011).

**Conclusion:**

Anteromedial bundle injury alters medial compartment loading during gait, causing a slight posterior shift of the center of CCS. And that anatomical single-bundle ACLR does not fully replicate the native anterior cruciate ligament’s biomechanical function.

## 1 Introduction

The anterior cruciate ligament (ACL) is a critical structure for maintaining knee joint stability, which consists of two distinct functional bundles: the anteromedial (AM) bundle and the posterolateral (PL) bundle (Petersen and Zantop, 2007). The AM bundle primarily resists anterior tibial translation during knee flexion, while the PL bundle provides rotational stability, particularly in extension (Tiamklang et al., 2012). Together, these bundles ensure dynamic stability during various activities, including walking. Partial injuries to the ACL, particularly those involving the AM bundle injury, account for approximately 10%–27% of cases (Colombet et al., 2010; Temponi et al., 2015) and can significantly disrupt joint mechanics (Chun et al., 2002). These injuries often present with subtle clinical symptoms, complicating early diagnosis and increasing the risk of progression to complete rupture and joint degeneration (DeFranco and Bach, 2009).

Although extensive research has been conducted on the biomechanics of complete ACL tears, partial injuries (Colombet et al., 2010), especially isolated AM bundle injuries, constitute a distinct clinical entity where the knee retains partial stability but undergoes biomechanical alterations during functional activities. Clinically, this condition may lead to compensatory changes in gait, altered load distribution across the tibiofemoral joint (Tiamklang et al., 2012), and an elevated risk of post-traumatic knee osteoarthritis (KOA) (DeFranco and Bach, 2009; Messner and Maletius, 1999). Understanding the mechanical consequences of partial injuries under dynamic loading conditions is essential for guiding treatment decisions—whether through conservative management or surgical reconstruction—and for developing strategies to prevent long-term joint degeneration.

Traditionally, biomechanical studies of ACL injuries have predominantly utilized static assessments, such as knee flexion (Liu et al., 2018; Marom et al., 2021; Nhan et al., 2021) and pivot-shift maneuvers (Marom et al., 2021), to investigate joint stability and load distribution. While these methods provide valuable insights, they fail to replicate the dynamic, weight-bearing conditions of normal walking—a fundamental activity of daily life. Furthermore, previous research has largely focused on changes in contact pressure (Liu et al., 2018; Marom et al., 2021) and contact area (Geeslin et al., 2016; LaPrade et al., 2014), while neglecting the anterior-posterior displacement of the stress center (contact center of stress, CCS) (Imhauser et al., 2016) as well as its spatial distribution across the joint. These dynamic parameters may be more indicative of functional alterations in knee biomechanics and could offer critical insights into the mechanisms underlying KOA development following ACL injuries.

To address these gaps, this study utilized a six-degree-of-freedom (6DOF) robotic system to simulate normal walking, driving cadaveric knee specimens through motion patterns based on the gait characteristics of humans (Bergmann et al., 2014). Pressure sensors placed beneath the menisci were employed to quantify the tibiofemoral joint’s pressure distribution and CCS displacement in three distinct states: intact ACL, AM bundle injury, and anatomical single-bundle reconstruction (Wu et al., 2010; Kondo et al., 2014). This innovative approach enabled a dynamic assessment of joint mechanics, providing a more realistic and clinically relevant understanding of ACL injury and reconstruction outcomes.

This study aims to quantify the changes in CCS displacement and pressure distribution across the tibiofemoral joint during simulated gait in the context of partial ACL injuries. By contrasting the intact, injured, and reconstructed states, this research seeks to elucidate the biomechanical alterations associated with AM bundle injuries and evaluate the efficacy of anatomical single-bundle reconstruction. We hypothesized that isolated AM bundle deficiency would disrupt the AP regional loading patterns of the knee, particularly during weight-bearing phases. Subsequently, we further posited that anatomic single-bundle ACL reconstruction (ACLR) might partially restore these altered stress patterns but would not fully replicate the intact knee’s biomechanical behavior. The findings will provide critical insights into how partial ACL injuries contribute to KOA development and offer evidence for optimizing clinical decision-making and rehabilitation strategies.

## 2 Methods

### 2.1 Specimen preparation

The study received approval from the Ethics Committee of Guangdong Provincial People’s Hospital (NO. 2019-226H-1) and the Department of Anatomy, School of Basic Medical Sciences, Southern Medical University. Seven fresh-frozen cadaveric knee specimens were obtained, with ages ranging from 31 to 47 years, comprising four males and three females. All Specimens were stored at −20°C and thawed overnight at room temperature. Each specimen underwent knee arthroscopy inspection prior to testing to confirm the absence of osteoarthritis, ACL injury, and any potential comorbidities that could affect the outcomes. The semitendinosus and gracilis tendons were harvested via an anteromedial incision at the proximal tibia for autologous ligament reconstruction. The dissection process involved the careful excision of skin and soft tissues, ensuring the preservation of crucial stabilizing structures, including the quadriceps, iliotibial tract, capsule, and both cruciate and collateral ligaments. The tibia and femur were transected at a distance of 15 cm from the joint line (Koh et al., 2018). The fibula aligned and was fixed to the tibia’s anatomical position utilizing a 2.5-mm Kirschner wire. Subsequently, using methyl methacrylate to affix the custom-fitted cylinders to the distal tibiofibular complex.

### 2.2 Intact knee joint testing

**FIGURE 1:**
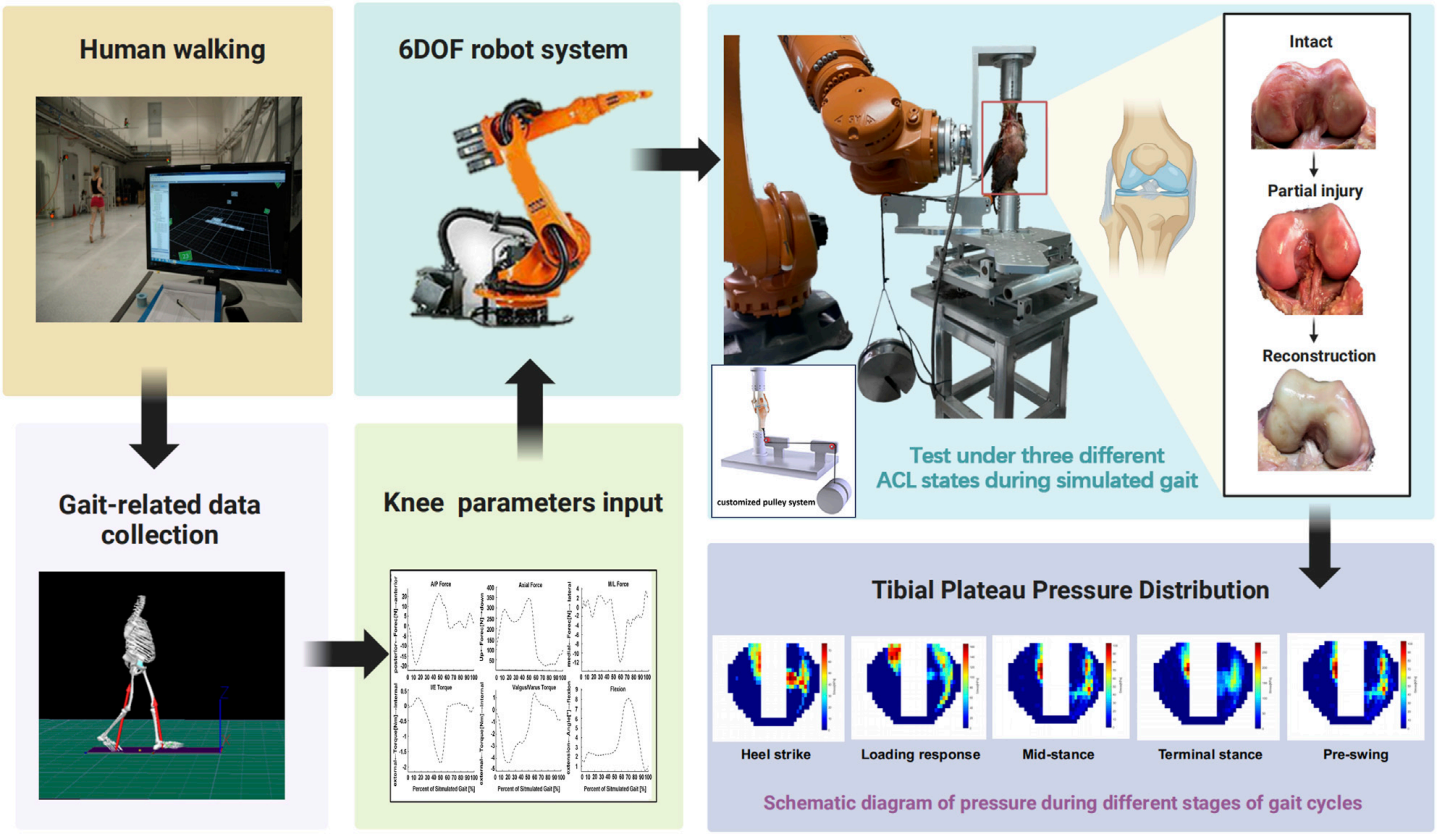
A diagram of the experimental procedure. (Figure was created with Biorender.com).

The specimen was secured to a multi-directional loading robotic testing system with six degrees of freedom, adhering to the protocol delineated by Woo et al. (2002) The KUKA AG KR 120 R2500 Pro (Augsburg, Germany), a 6DOF robotic system (Figure 1), exhibits a joint motion repeatability of ±0.06 mm. Complementing this system is the load cell (Model FT Delta, ATI Industrial Automation, Apex, NC, United States, which offers a force accuracy of ±0.2 N and a moment accuracy of ±0.1 N m. A custom MATLAB program, running on a multitasking operating system (MathWorks Inc., Natick, MA, United States), was employed to control the knee kinematic parameters, guaranteeing robust test-retest reliability (Papageorgiou et al., 2001; Sakane et al., 1997; Nakamura et al., 2019; Cheng et al., 2024) (Figure 1). The robotic testing system was controlled under both displacement and force control modes. To ascertain the six-degree-of-freedom path of passive knee flexion-extension, the intact cadaveric knee underwent passive flexion from 0° to 90°. Firstly, the system determined the position of the knee joint at every degree of flexion increment and ensured minimized external forces and torques. Then, the knee was unloaded internally at each position, provided as both reference points for the measure of kinematic parameters and the initial point for external loads throughout the testing process (Sakane et al., 1997). A 10 kg weight was suspended from one end of a rope attached to the customized pulley system, while the other end was sutured to the quadriceps tendon. By passing the rope through two pulleys (as shown in Figure 1), the 100 N gravitational force of the weight was applied to the quadriceps tendon. The force direction was carefully aligned with the natural orientation of the tendon, effectively simulating quadriceps contraction and its loading effect on the knee joint. This setup was designed to compensate for the loss of residual muscular support caused by femoral transection, thereby preserving joint stability (Yoo et al., 2005; Huang et al., 2021; Markolf et al., 2004).

### 2.3 Contact stress measurement

To assess contact mechanics, four 1.5 cm submeniscal incisions were made on both the anterior and posterior aspects of the lateral and medial menisci. A Tekscan stress sensor (Model 4,010, Tekscan Inc., Boston, MA, United States) was inserted appropriately beneath the menisci via incisions, consistent with established methods (Liu et al., 2018; Logan et al., 2019). The sensor system comprises two independent grids of sensing elements, designed for insertion into the medial and lateral compartments of the knee joint. Each sensor has a measurement area of 1.9 mm × 1.9 mm. The medial and lateral grids are structured with a maximum of 21 rows, corresponding to a length of 40.0 mm, and up to 13 columns, spanning a width of 24.8 mm. This configuration enables precise measurement of pressure distribution across the tibiofemoral joint compartments. The sensor tabs were sutured close to the ACL insertion site and in the posterior capsule, respectively. Before implantation, based on the manufacturer’s recommendation and guideline, the calibration procedures were performed at maximum axial loading force during the gait cycle, with an accuracy within 25 N (approximately 5%) of the calibrated load, and a repeatability within 1% (Bergmann et al., 2014; LaPrade et al., 2014).

### 2.4 Dynamical simulated human gait

We used the real-world human mechanical parameters from the Orthoload database to simulate human gait motion (https://orthoload.com/), including axial force, medial-lateral force, anterior-posterior force, valgus-varus torque, and internal-external torque (Bergmann et al., 2014). Additionally, all degrees of freedom were governed by force-control mode, except for the prescribed flexion angle (Figure 1).

### 2.5 Testing conditions

Three conditions of ligament status were tested sequentially (Petersen and Zantop, 2007): intact ACL (INT) (Tiamklang et al., 2012), anteromedial bundle deficiency (AMD), and (Colombet et al., 2010) anatomical single-bundle ACL reconstruction (ACLR). A skillful orthopedic surgeon carried out all the surgical procedures. The testing procedures were in a stepwise manner. First, calibrate and measure the results on the intact knee joint as the baseline. Subsequently, an orthopaedic surgeon identified the AM and PL bundles via medial arthrotomy, with verification by another surgeon. Using curved forceps to carefully separate the AM and PL bundles and resect the AM bundles using a No. 11 scalpel, including stump of the femoral and tibia insertion, consistent with previous studies (Wu et al., 2010; Kondo et al., 2014). After that, careful attention was paid to repairing joint cavities and other structures.

In the last test condition, based on the internationally recognized anatomical single-bundle ACL reconstruction technique, following the guidelines of the American Academy of Orthopaedic Surgeons (AAOS), this study utilized a quadrupled autograft of the semitendinosus and gracilis tendons (7.5–8 mm in diameter) for single-bundle reconstruction (Mao et al., 2020; Morimoto et al., 2009). To achieve dual stability, fixation was performed using a combination of an interface screw within the bone tunnel and an Endobutton suspension system. The femoral tunnel was positioned with the posterior-superior corner of the lateral femoral condyle as the entry point and the midpoint of the posterior wall of the intercondylar notch as the exit point. The tibial tunnel was established with the midpoint between the tibial crest and the medial tibial border as the entry point and centered at the anatomical footprints of the anteromedial and posterolateral bundles of the ACL as the exit point (Fox et al., 2023). The graft tensioning protocol followed a standardized approach, with the graft tensioned to 80 N at 20°–30° of knee flexion, followed by sequential fixation, first at the femoral side and then at the tibial side, with repeated knee flexion-extension cycles before final fixation to optimize graft adaptation and minimize postoperative laxity. On the femoral side, an Endobutton suspension fixation system (Smith and Nephew Endoscopy) was employed, with the appropriate Endobutton size selected based on graft length to ensure at least 20 mm of effective graft length retained within the femoral tunnel. On the tibial side, fixation was performed at 30° of knee flexion using a polyether-ether-ketone (PEEK) interference screw (Suzhou Suke Medical Instruments Co., Ltd., Changzhou, China) to enhance stability and reduce tunnel widening (Figure 1).

Based on the classification methodologies, knee kinematics and contact parameters were calculated at five distinct points of the simulated gait cycle: 2% (flexion angle:3.7°), 12% (flexion angle:9.2°), 30% (flexion angle:7.8°), 50% (flexion angle:11.2°), and 60% (flexion angle:32.2°). These carefully chosen instances correspond to crucial events throughout the stance phase, namely, heel strike (2%), loading response (12%), mid-stance (28%), terminal stance (50%), and pre-swing (60%) (Moltedo et al., 2018).

**FIGURE 2:**
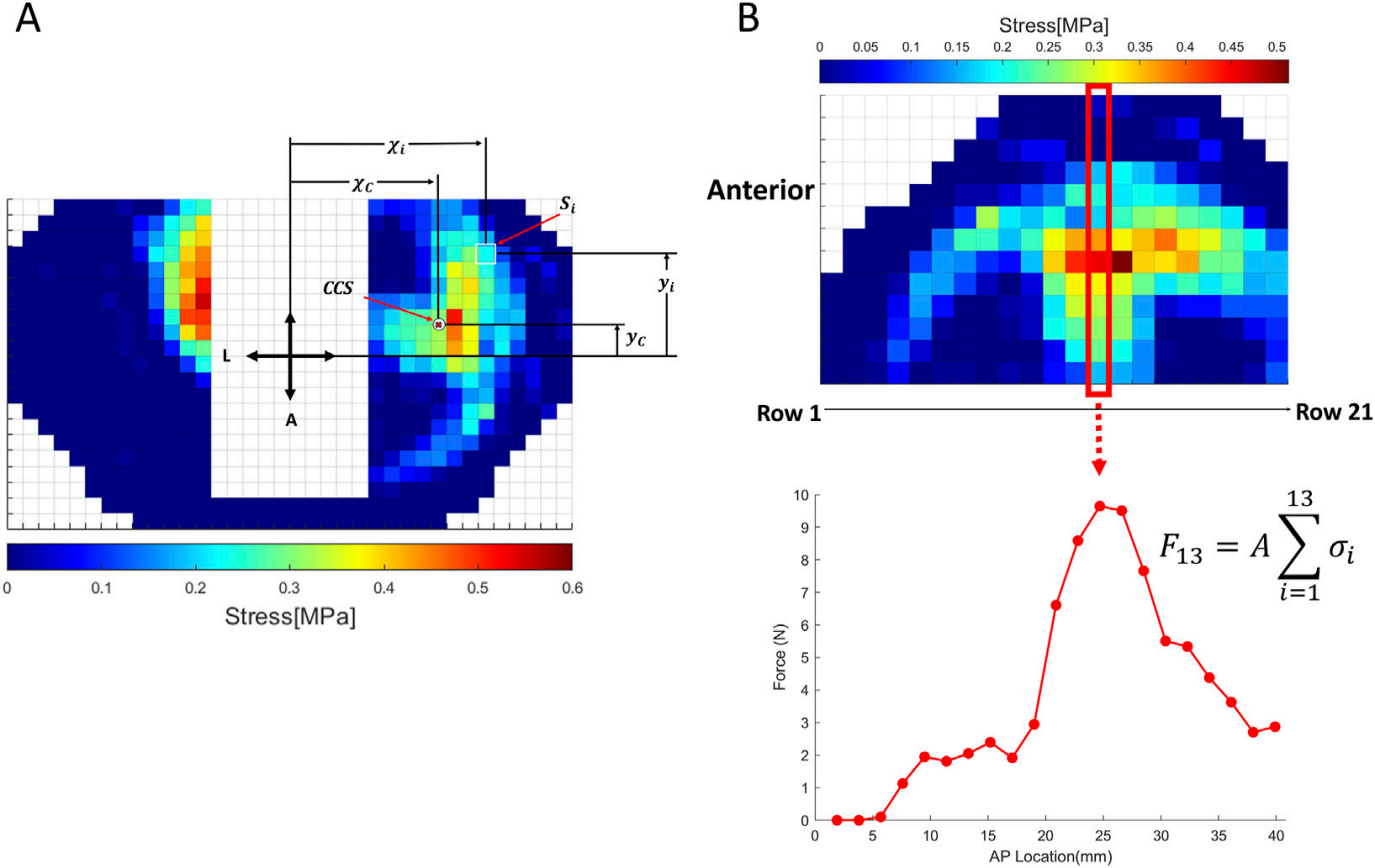
Subplot **(A)** Illustration of contact center of stress (CCS) and calculation method; Subplot **(B)** Illustration of regional loading calculation and calculation method.

We computed the magnitude of peak contact stress, CCS and anterior-posterior (AP) regional loading (definition and explanation in the “Data Analysis” section and in Figure 2B) on the lateral/medial tibial plateau by replicating the testing under successive conditions.

### 2.6 Data analysis

The primary outcome of this study was the AP displacement of the CCS in the medial and lateral compartments of the tibiofemoral joint. This parameter is a crucial indicator of knee joint stability and functional status. By quantifying shifts in the pressure center, it is possible to evaluate alterations in load distribution and mechanical alignment under different ligamentous conditions: intact ACL, AM bundle injury, and anatomical single-bundle reconstruction. This study utilized seven cadaveric knee specimens, a sample size that achieves a balance between statistical rigor and the practical limitations inherent to cadaveric biomechanical research. The inclusion of multiple specimens accounts for variability in joint anatomy and ligament properties, ensuring repeatability while aligning with prior studies (Marom et al., 2021; LaPrade et al., 2015) that have demonstrated the reliability of small cohorts for investigating joint mechanics.

The location of the CCS ( $x_{C},y_{C}$ ) (Imhauser et al., 2016) in the medial and lateral compartments was calculated using a weighted average of the contact stress values ( $S_{i}$ ) recorded by individual sensing elements (sensels). The calculation was performed as follows (Figure 2A):

$$x_{C}=\frac{\sum_{i=1}^{n}x_{i}S_{i}}{\sum_{i=1}^{n}S_{i}},y_{C}=\frac{\sum_{i=1}^{n}y_{i}S_{i}}{\sum_{i=1}^{n}S_{i}},$$

Here, $x_{i}$ and $y_{i}$ represent the coordinates of the $i^{th}$ sensing element, $S_{i}$ ​ is the corresponding contact stress, and n is the total number of sensing elements. These calculations were performed separately for the medial and lateral compartments at each step of the gait simulation.

The AP displacement of the CCS was determined by tracking changes in $x_{C}$ ​ (the anterior-posterior coordinate) throughout the simulated gait cycle. Displacement values were expressed relative to the intact ACL condition to evaluate the effects of AM bundle injury and reconstruction. Positive and negative shifts in $x_{C}$ indicated anterior and posterior movements of the stress center, respectively.

Regional AP loading (Marom et al., 2021) was defined as the total force transmitted through each row of sensels in the AP direction, providing a detailed profile of load distribution along the tibiofemoral joint. The total force ( $F_{k}$ ) acting on row k was calculated as (Figure 2B):

$$F_{k}=A\cdot \sum_{i=1}^{m}\sigma_{i}$$

where A is the area of each sensel, m is the number of sensels in row k , and $\sigma_{i}$ is the contact stress recorded by the $i^{th}$ sensel in that row. Regional loading profiles were analyzed for both the medial and lateral compartments under each ligamentous condition to assess changes in force transmission patterns.

This comprehensive data analysis approach allowed for detailed biomechanical assessment of the knee joint, providing insights into how AM bundle injuries and single-bundle ACL reconstruction influence tibiofemoral pressure distribution and joint stability during gait simulation.

### 2.7 Statistical analysis

The statistical analyses were conducted using SPSS Statistics version 22.0 (IBM Corp., Armonk, NY, United States) and MATLAB (MathWorks Inc.). To compare the AP displacement of the CCS relative to intact knee between the AMD and ACLR, paired t-tests were applied for normally distributed data. For non-normal data, the Wilcoxon signed-rank test was used. A repeated-measures analysis of variance (ANOVA) was used to evaluate differences in the peak contact pressure, and regional AP loading across the three conditions (intact ACL, AMD, and ACLR). For variables that did not follow a normal distribution, the Wilcoxon matched-pairs signed-rank test is employed. Post hoc comparisons were conducted with Bonferroni adjustment if there were significant overall differences, with a significance level set at p < 0.05.

## 3 Results

### 3.1 Peak contact stress

Table 1 summarizes the peak contact stress values for INT, AMD, and ACLR knees across five gait cycle stages. In the lateral compartment, AMD knees exhibited no significant differences from INT knees during these stages. At 2% of the gait cycle, ACLR knees exhibited a peak contact stress (0.21 ± 0.15 MP) slightly higher than INT knees (0.12 ± 0.16 MP). No significant differences were observed in the lateral compartment at other stages (p > 0.05); In the medial compartment, both AMD and ACLR knees exhibited no significant differences from INT knees during all of the five stages.

**TABLE 1:** Peak contact stress (MPa) of the INT, AMD, and ACLR Knee in response to five stages of the gait cycle.

Gait cycle stage	Lateral			Medial		
	INT	AMD	ACLR	INT	AMD	ACLR
2%	0.12 ± 0.16	0.18 ± 0.21	0.21 ± 0.15*	0.47 ± 0.29	0.48 ± 0.29	0.54 ± 0.37
12%	0.49 ± 0.56	0.59 ± 0.75	0.57 ± 0.51	0.5 ± 0.34	0.48 ± 0.37	0.48 ± 0.38
30%	0.45 ± 0.64	0.53 ± 0.74	0.52 ± 0.46	0.48 ± 0.45	0.46 ± 0.44	0.5 ± 0.54
50%	0.59 ± 0.93	0.66 ± 0.9	0.65 ± 0.58	0.87 ± 0.49	0.8 ± 0.51	1.03 ± 0.62
60%	0.25 ± 0.08	0.3 ± 0.16	0.49 ± 0.43	0.45 ± 0.35	0.38 ± 0.29	0.56 ± 0.51

The data are presented as the mean ± standard deviation.

*P < .05 compared with the intact knee.

INT, anterior cruciate ligament intact; AMD, anterior cruciate ligament anteromedial deficiency; ACLR, anterior cruciate ligament reconstruction.

### 3.2 AP displacement of the CCS

**FIGURE 3:**
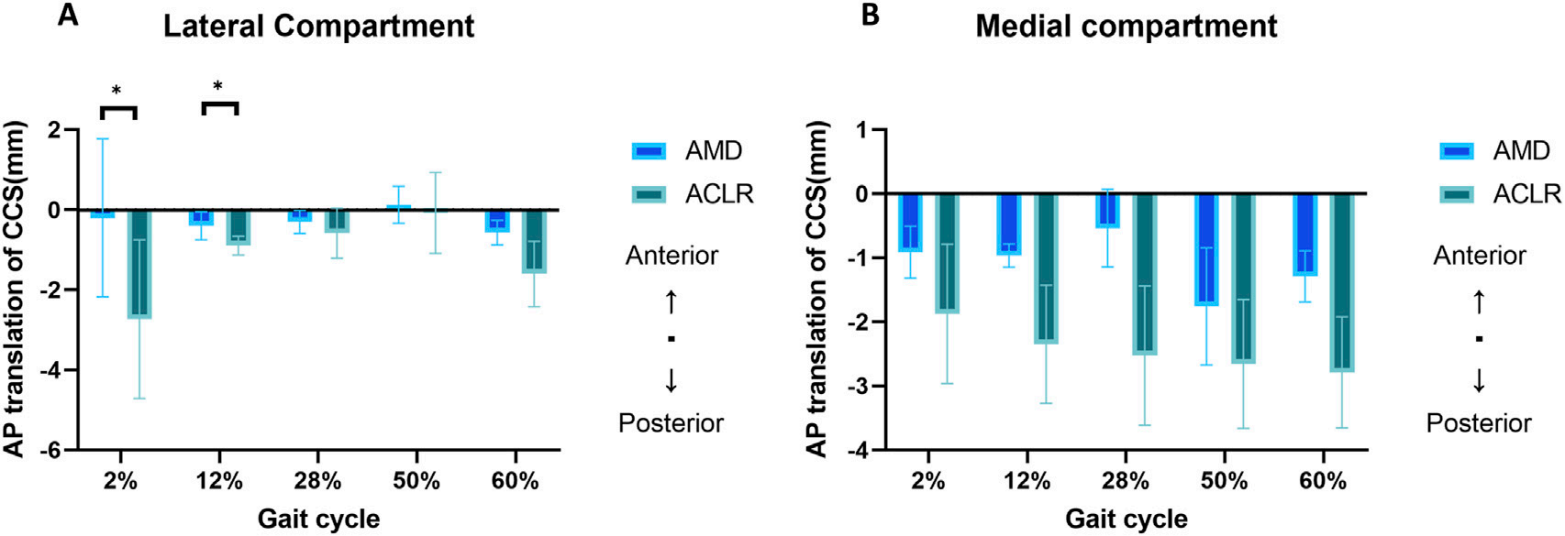
Mean changes (mm) in AP location of CCS of AMD and ACLR knees relative to the INT knee in response to five stages of gait cycle in the lateral compartment (plot A) and medial compartment (plot B). AP, anterior-posterior; CCS, contact center of stress; INT, intact knee; AMD, anteromedial bundle deficiency knee; ACLR, anterior cruciate ligament reconstruction knee. *: *P* < 0.05 between AMD and ACLR.

The mean changes in the AP location of the CCS in the lateral and medial compartments of AMD and ACLR knees relative to the INT knee across five gait cycle stages are presented in Figure 3. No significant differences were found at all the five gait cycle stages (p > 0.05) in the medial compartment; In the lateral compartment, a significant posterior displacement of the CCS was observed in ACLR knees compared to AMD knees at both the heel strike stage (0.21 ± 1.97 mm vs 2.73 ± 1.98mm, p = 0.022) and loading response stage (0.4 ± 0.35 mm vs. −0.89 ± 0.24  mm, p = 0.026). No significant differences were found at other gait cycle stages (p > 0.05) in the lateral compartment. While significant differences between AMD and ACLR conditions were limited to the early stance phase (2%–12% of the gait cycle), notable trends in AP displacement were observed across all stages. Both AMD and ACLR knees generally exhibited posterior shifts in CCS relative to the INT knee, particularly during the early stance phase. However, the magnitude of these shifts varied, with ACLR knees tending to show larger posterior displacements than AMD knees. The detail data are showed in Table 2.

**TABLE 2:** Mean changes (mm) in AP location of CCS of AMD and ACLR knees relative to the INT knee in response to five stages.

Gait cycle stage	Lateral				Medial			
	AMD	ACLR	Difference	p value	AMD	ACLR	Difference	p value
2%	−0.21 ± 1.97	−2.73 ± 1.98	2.53	**0.022**	−0.91 ± 0.41	−1.87 ± 1.09	0.96	0.445
12%	−0.4 ± 0.35	−0.89 ± 0.24	0.49	**0.026**	−0.97 ± 0.18	−2.35 ± 0.92	1.38	0.191
28%	−0.3 ± 0.29	−0.58 ± 0.63	0.28	0.523	−0.54 ± 0.61	−2.53 ± 1.09	1.99	0.194
50%	0.13 ± 0.46	−0.08 ± 1.01	0.21	0.793	−1.76 ± 0.91	−2.66 ± 1	0.9	0.086
60%	−0.57 ± 0.31	−1.6 ± 0.82	1.03	0.251	−1.29 ± 0.4	−2.79 ± 0.87	1.5	0.095

The data are presented as the mean ± standard deviation.

The red font indicates a significant difference between AMD, and ACLR.

AP, anterior-posterior; CCS, center of contact stress; INT, anterior cruciate ligament intact; AMD, anterior cruciate ligament anteromedial deficiency; ACLR, anterior cruciate ligament reconstruction.

### 3.3 Regional loading of the lateral compartment

#### 3.3.1 AMD knees

**FIGURE 4:**
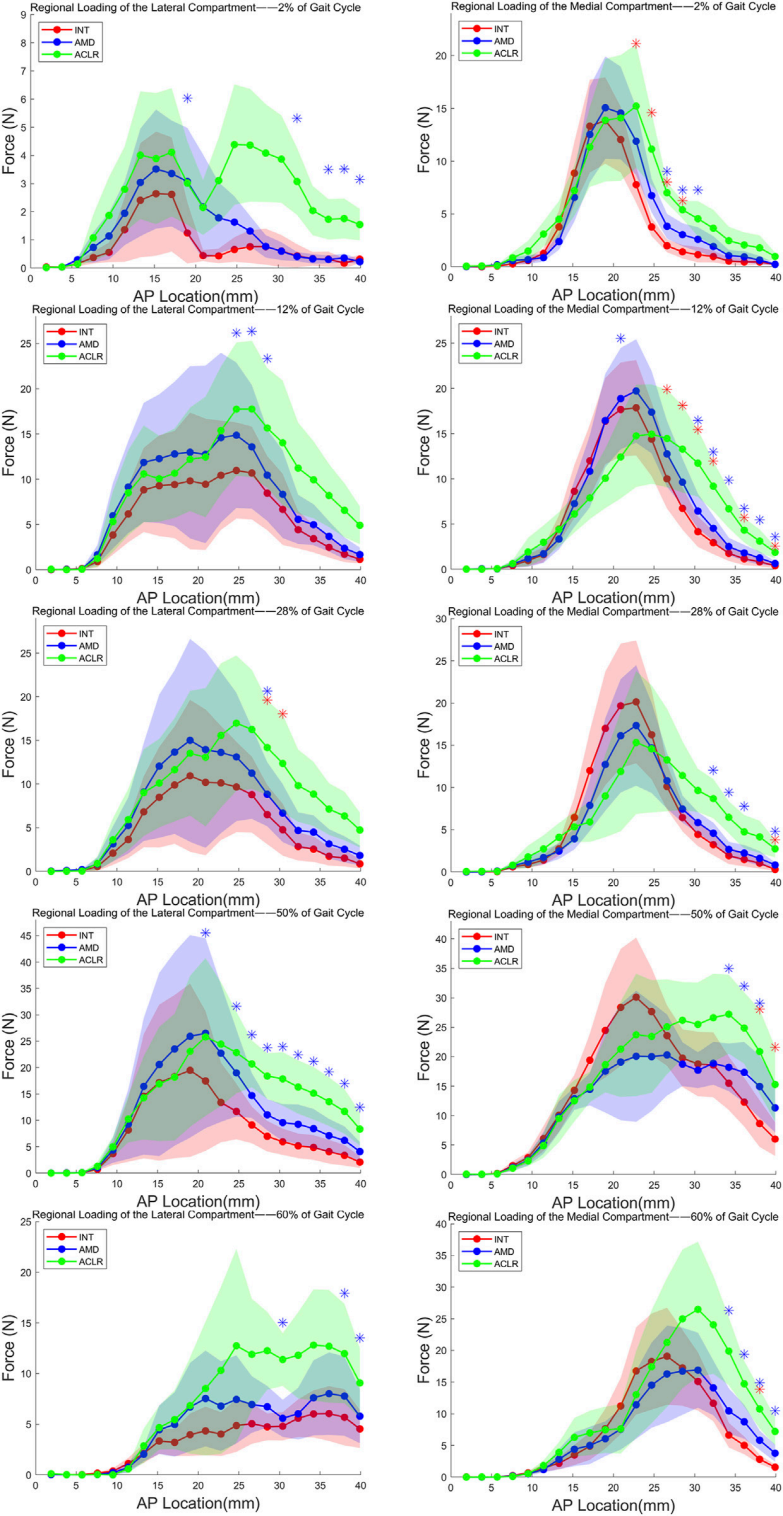
Loading of the both compartments from the most anterior (left) to the most posterior (right) row of the stress transducer in response to five stages of gait cycle. AP, anterior-posterior; INT, intact knee; AMD, anteromedial bundle deficiency knee; ACLR, anterior cruciate ligament reconstruction knee. Red *: *P* < 0.05 between INT and AMD, Blue *: *P* < 0.05 between INT and ACLR.

Compared to the intact condition, the lateral compartment of AMD knees exhibited a significant increase in contact force during the mid-stance phase (28% of the gait cycle) as showed in Figure 4; Table 3. While contact forces in the lateral compartment were elevated during other phases of the gait cycle relative to intact knees, these changes were not statistically significant, and no notable alterations in force distribution were observed. Specifically, during mid-stance, the lateral compartment of AMD knees demonstrated a significant increase in contact force over a 3.8 mm-wide region (comprising 2 rows of the stress transducer). The most substantial force increase acting on any of these rows was 2.33 ± 0.29 N (p = 0.049).

**TABLE 3:** Regional Loading(N) of the lateral and medial compartment from the most anterior to the most posterior row of the stress transducer.

Gait cycle stages	Rows of the stress transducer	Region of compartment (mm)	Lateral					Medial				
			INT	AMD	ACLR	p value_1	p value_2	INT	AMD	ACLR	p value_1	p value_2
2%	10	19	1.24 ± 1.08	3.07 ± 1.9	3.02 ± 1.48	0.325	**0.032**	13.84 ± 4.09	15.07 ± 4.84	13.88 ± 5.8	0.371	0.991
	12	22.8	0.43 ± 0.26	1.78 ± 1.26	3.11 ± 1.52	0.331	0.142	7.78 ± 1.71	11.89 ± 3.2	15.22 ± 5.85	**0.041**	0.177
	13	24.7	0.66 ± 0.43	1.63 ± 0.92	4.38 ± 2.14	0.341	0.136	3.77 ± 0.87	6.74 ± 1.59	11.14 ± 3.42	**0.024**	0.097
	14	26.6	0.76 ± 0.58	1.31 ± 0.54	4.36 ± 2	0.457	0.129	1.99 ± 0.61	3.83 ± 1.17	7.01 ± 0.97	**0.041**	**0.014**
	15	28.5	0.76 ± 0.64	0.77 ± 0.4	4.08 ± 1.74	0.989	0.101	1.41 ± 0.64	3.04 ± 1.08	5.39 ± 0.83	**0.042**	**0.008**
	16	30.4	0.6 ± 0.56	0.6 ± 0.32	3.87 ± 1.55	0.988	0.064	1.14 ± 0.74	2.59 ± 1.15	4.55 ± 1.68	0.063	**0.034**
	17	32.3	0.43 ± 0.41	0.41 ± 0.17	3.07 ± 1.19	0.95	**0.036**	0.97 ± 0.56	1.92 ± 0.98	3.65 ± 1.75	0.166	0.098
	19	36.1	0.31 ± 0.27	0.3 ± 0.14	1.73 ± 0.72	0.963	**0.041**	0.45 ± 0.36	0.93 ± 0.54	2.07 ± 1.18	0.112	0.109
	20	38	0.17 ± 0.17	0.35 ± 0.16	1.76 ± 0.71	0.24	**0.041**	0.45 ± 0.23	0.62 ± 0.4	1.79 ± 1.18	0.504	0.256
	21	39.9	0.31 ± 0.15	0.22 ± 0.07	1.54 ± 0.56	0.403	**0.031**	0.22 ± 0.11	0.22 ± 0.12	0.97 ± 0.37	0.993	0.099
12%	11	20.9	9.44 ± 7.24	12.75 ± 9.81	12.45 ± 6.66	0.263	0.159	17.67 ± 5.2	18.87 ± 5.6	12.41 ± 4.59	0.311	**0.044**
	13	24.7	10.97 ± 5.31	14.86 ± 8.03	17.75 ± 7.37	0.23	**0.039**	14.38 ± 4.08	17.37 ± 4.55	14.94 ± 5.5	0.111	0.856
	14	26.6	10.69 ± 5.13	13.57 ± 6.83	17.76 ± 7.55	0.207	**0.046**	10 ± 3.29	12.75 ± 3.64	14.46 ± 5.39	**0.019**	0.171
	15	28.5	8.46 ± 4.09	10.45 ± 5.05	15.66 ± 6.62	0.146	**0.041**	6.73 ± 2.34	9.63 ± 2.61	13.28 ± 4.8	**0.016**	0.083
	16	30.4	6.66 ± 3.92	8.34 ± 4.83	14.02 ± 6.88	0.121	0.062	4.15 ± 1.68	6.43 ± 1.92	11.72 ± 3.69	**0.007**	**0.021**
	17	32.3	4.42 ± 2.06	5.57 ± 2.71	11.23 ± 5.03	0.139	0.074	2.94 ± 1.28	4.53 ± 1.4	9.18 ± 2.75	**0.01**	**0.022**
	18	34.2	3.43 ± 1.53	4.98 ± 2.26	9.94 ± 4.26	0.084	0.068	1.76 ± 0.84	2.51 ± 0.97	6.68 ± 2.12	0.11	**0.033**
	19	36.1	2.47 ± 1.13	3.68 ± 1.68	8.19 ± 3.48	0.09	0.069	1.16 ± 0.7	1.81 ± 0.89	4.32 ± 1.35	**0.026**	**0.014**
	20	38	1.71 ± 0.87	2.37 ± 1.2	6.56 ± 2.99	0.108	0.091	0.82 ± 0.5	1.24 ± 0.72	3.11 ± 1.29	0.13	**0.044**
	21	39.9	1.15 ± 0.44	1.67 ± 0.78	4.91 ± 2.08	0.221	0.088	0.4 ± 0.23	0.64 ± 0.23	1.87 ± 0.65	**0.038**	**0.028**
28%	15	28.5	6.5 ± 3.38	8.83 ± 3.67	14.18 ± 5.39	**0.05**	**0.043**	6.44 ± 1.63	7.44 ± 1.61	11.41 ± 4.07	0.524	0.23
	16	30.4	4.77 ± 2.97	6.67 ± 3.31	12.36 ± 5.61	**0.045**	0.068	4.43 ± 1.38	5.84 ± 1.25	9.64 ± 2.56	0.36	0.081
	17	32.3	2.84 ± 1.6	4.67 ± 2.08	9.82 ± 4.13	0.077	0.08	3.23 ± 1.25	4.58 ± 1.26	8.68 ± 2.32	0.291	**0.026**
	18	34.2	2.54 ± 1.46	4.49 ± 1.92	8.86 ± 3.68	0.076	0.085	1.92 ± 0.98	2.66 ± 0.91	6.47 ± 1.89	0.301	**0.009**
	19	36.1	1.73 ± 1.06	3.15 ± 1.67	7.13 ± 2.95	0.096	0.081	1.48 ± 0.82	2.23 ± 0.96	4.75 ± 1.99	0.168	**0.04**
	21	39.9	0.86 ± 0.52	1.82 ± 1.14	4.73 ± 1.99	0.192	0.065	0.29 ± 0.16	0.83 ± 0.29	2.74 ± 1.02	**0.039**	**0.039**
50%	11	20.9	17.47 ± 13.25	26.5 ± 17.99	25.8 ± 14.96	0.135	**0.031**	28.37 ± 9.93	19.09 ± 9.88	21.28 ± 8.13	0.227	0.299
	13	24.7	11.69 ± 5.26	19 ± 8.23	22.85 ± 7.73	0.146	**0.03**	27.65 ± 7.4	20 ± 9.2	23.43 ± 9.57	0.256	0.493
	14	26.6	9.12 ± 3.47	14.7 ± 4.24	20.7 ± 4.46	0.157	**0.018**	23.58 ± 5.12	20.29 ± 6.88	25.08 ± 8.05	0.508	0.8
	15	28.5	6.98 ± 2.89	11.06 ± 2.95	18.39 ± 4.33	0.163	**0.017**	19.76 ± 4.89	18.74 ± 3.72	26.19 ± 6.49	0.816	0.325
	16	30.4	5.95 ± 2.49	9.57 ± 3.49	17.85 ± 5.07	0.246	**0.027**	18.76 ± 5.47	17.71 ± 3.04	25.49 ± 7.13	0.745	0.17
	17	32.3	5.16 ± 2.33	9.25 ± 3.75	16.31 ± 5.05	0.206	**0.033**	18.6 ± 5.52	18.88 ± 3.64	26.62 ± 7.46	0.92	**0.05**
	18	34.2	4.86 ± 2.34	8.43 ± 3.73	15.14 ± 4.98	0.21	**0.035**	15.49 ± 4.54	18.2 ± 4.05	27.22 ± 6.71	0.435	**0.011**
	19	36.1	4.06 ± 2.25	7.11 ± 3.16	13.53 ± 4.6	0.211	**0.036**	12.28 ± 4.14	17.34 ± 5.15	24.86 ± 6.06	0.112	**0.004**
	20	38	3.36 ± 1.95	6.2 ± 2.65	11.7 ± 4.24	0.135	**0.043**	8.63 ± 3.96	14.94 ± 5.3	20.87 ± 7.17	**0.049**	**0.032**
	21	39.9	2.08 ± 1.1	4.08 ± 1.83	8.34 ± 3.1	0.155	**0.046**	6.01 ± 2.85	11.31 ± 4.18	15.29 ± 6.29	**0.043**	0.078
60%	16	30.4	4.8 ± 1.39	5.58 ± 1.83	11.38 ± 2.6	0.516	**0.016**	15.11 ± 4.59	16.89 ± 5.92	26.48 ± 10.68	0.323	0.192
	18	34.2	6.02 ± 2.33	7.6 ± 3.71	12.81 ± 5.5	0.343	0.08	6.63 ± 1.96	10.45 ± 3.56	19.89 ± 5.38	0.119	**0.042**
	19	36.1	6.05 ± 2.71	8.02 ± 4.07	12.69 ± 5.55	0.223	0.06	5 ± 1.4	8.73 ± 2.68	14.73 ± 3.61	0.071	**0.014**
	20	38	5.68 ± 2.78	7.77 ± 3.91	11.97 ± 4.91	0.144	**0.04**	2.81 ± 0.74	5.81 ± 1.53	10.75 ± 3.09	**0.044**	**0.026**
	21	39.9	4.54 ± 1.88	5.79 ± 2.67	9.08 ± 3.4	0.266	**0.032**	1.56 ± 0.39	3.77 ± 1.35	7.21 ± 2.21	0.072	**0.03**

The data are presented as the mean ± standard deviation.

INT, anterior cruciate ligament intact; AMD, anterior cruciate ligament anteromedial deficiency; ACLR, anterior cruciate ligament reconstruction.

p value_1:INT, vs AMD; p value_2:INT, vs ACLR; the red font indicates a significant difference.

In the medial compartment, AMD knees exhibited significant differences across five phases of the gait cycle. Notably, during heel strike (2% of the gait cycle), loading response (12%), and terminal stance (50%), multiple continuous points of significant force increase were observed. These phases corresponded to regions of significant contact force increases measuring 7.6 mm (comprising 4 rows of the stress transducer), 11.4 mm (comprising 6 rows), and 3.8 mm (comprising 2 rows), respectively. The greatest force increases acting on any of these rows were 4.11 ± 1.5 N (p = 0.04), 2.9 ± 0.27 N (p = 0.016), and 6.31 ± 1.35 N (p = 0.048), respectively. Although the force increases were significant across multiple phases, the overall magnitude of change was modest, and there were no significant shifts in force distribution.

#### 3.3.2 ACLR knees

In the lateral compartment, ACLR knees showed significant differences across five phases of the gait cycle (Figure 4). Particularly, during heel strike (2% of the gait cycle), loading response (12%), terminal stance (50%), and pre-swing (60%), multiple continuous points of significant force increase were observed. These phases corresponded to regions of significant contact force increases measuring 9.5 mm (comprising 5 rows of the stress transducer), 5.7 mm (comprising 3 rows), 19 mm (comprising 10 rows), and 5.7 mm (comprising 3 rows), respectively. The greatest force increases acting on any of these rows were 2.64 ± 0.78 N (p = 0.036), 7.2 ± 2.53 N (p = 0.041), 11.91 ± 2.58 N (p = 0.027), and 6.59 ± 1.21 N (p = 0.016), respectively. Compared to the intact condition, ACLR knees demonstrated force increases of varying magnitudes across the five stance phases, along with a discernible posterior shift in force distribution.

In the medial compartment, ACLR knees also exhibited significant differences across all five stance phases, with multiple continuous points of force increase identified. These phases corresponded to regions of significant contact force increases measuring 5.7 mm (comprising 3 rows of the stress transducer), 13.3 mm (comprising 7 rows), 7.6 mm (comprising 4 rows), 5.7 mm (comprising 3 rows), and 7.6 mm (comprising 4 rows), respectively. The greatest force increases acting on any of these rows were 5.02 ± 0.36 N (p = 0.014), 7.57 ± 2.02 N (p = 0.021), 5.45 ± 1.08 N (p = 0.026), 11.72 ± 2.17 N (p = 0.011), and 13.26 ± 3.43 N (p = 0.042), respectively. Similar to the lateral compartment, ACLR knees in the medial compartment showed varying degrees of force increase across all five stance phases, with a notable posterior shift in force distribution.

## 4 Discussion

This study aimed to investigate the changes in intra-articular contact mechanics during gait following AM bundle injury of the ACL and to evaluate whether anatomical single-bundle ACL reconstruction could restore these altered mechanics. Unlike previous studies focusing primarily on static conditions, such as knee flexion or pivot-shift tests, our investigation emphasized anterior-posterior displacement of the center of contact stress and regional loading changes throughout the gait cycle. Contrary to our hypothesis, our principal finding was that AMD altered medial compartment regional loading compared to intact knees and that anatomical single-bundle ACLR failed to restore these changes, instead exacerbating the alterations.

AMD caused a slight posterior shift (Figure 3) of the CCS during the stance phase, with minimal changes in peak contact stress across compartments. However, regional loading increased primarily in the medial compartment during multiple stages of the gait cycle. This posterior shift aligns with prior studies suggesting that increased anterior tibial translation (ATT) contributes to posterior CCS displacement. For example, Cone et al. observed no significant ATT in porcine specimens with AMD at 40° and 90° of flexion, although complete ACL rupture resulted in pronounced ATT (Cone et al., 2020). Similarly, Lintner et al. reported no significant differences in ATT in human knees with AMD compared to intact knees when subjected to a 30-pound anterior load at 30° of flexion (Lintner et al., 1995). However, other studies have noted increased anterior laxity following partial ACL injury. For instance, Koo et al., using musculoskeletal simulations, demonstrated that reducing ACL stiffness by 75% led to a significant increase in ATT (Koo et al., 2023). Nhan et al. reported a 4 mm increase in ATT at 30° of flexion in cadaveric knees following AM bundle transection (Nhan et al., 2021). These inconsistencies likely stem from variations in experimental approaches, including differences in specimen sources (cadaveric, computational, or *in vivo* imaging), simulated activities (walking, pivot-shift, or specific flexion angles), and loading conditions (axial forces, varus-valgus moments, etc.).

Our findings suggest that while AMD introduces modest alterations to the biomechanical environment, its overall impact on the loading patterns during walking is limited. The slight posterior shift of the contact center of stress (CCS) may be due to a mild reduction in anteroposterior stability following anteromedial bundle injury, leading to a shift in the contact region between the tibial plateau and femoral cartilage. The absence of a more pronounced posterior shift is likely attributed to the compensatory function of other knee structures, such as the posteromedial structures, posterior cruciate ligament, and lateral collateral ligament. However, the observed increase in medial compartment regional loading might result from rotational shifts between the femur and tibia. This is consistent with findings by Jayson et al., who demonstrated that rotational laxity progressively increases in knees with partial and complete ACL tears (Lian et al., 2020). The absence of significant changes in peak contact stress in this study further suggests that isolated AMD may not impose excessive mechanical stress during walking.

Compared to the intact knee, ACLR knees exhibit a posterior shift of the CCS exceeding 2 mm during walking, along with increased peak pressure and a posterior shift in regional loading. Currently, research on CCS in the knee joint is limited, and no clear clinical consensus exists regarding the threshold for instability. However, Caroline et al. (Brial et al., 2019) reported that following lateral meniscectomy, the CCS shifted posteriorly by approximately 3 mm during the stance phase of gait, using the intact meniscus as a baseline. Similarly, Imhauser et al. (2016) found in an *in vitro* study that ACL-deficient knees exhibited a posterior CCS shift of approximately 3 mm during pivot-shift testing compared to the ACL-intact condition. In both cases—whether due to total meniscectomy or ACL deficiency—the knee was in an unstable state. Thus, a 3 mm posterior shift in CCS may serve as a potential threshold for defining instability. Based on this, we propose that while ACL reconstruction restricts excessive anterior tibial translation, it does not fully restore the CCS position to that of the intact knee during dynamic movement.

The inability of single-bundle ACL reconstruction to fully restore native knee biomechanics may stem from both graft property limitations and surgical technique constraints. The observed increase in regional loading and lateral compartment peak contact stress may be attributed to the increased graft stiffness in *ex vivo* experiments. Factors such as dehydration, the absence of blood supply, and cellular inactivity contribute to enhanced collagen cross-linking, which, in turn, increases ligament rigidity and leads to a more concentrated distribution of joint stress. To mitigate this effect, all ligament specimens were stored at −20°C immediately after extraction and rehydrated in phosphate-buffered saline (pH 7.4) for 2 hours before testing. Additionally, the increased laxity observed in ACLR knees may be influenced by surgical technique limitations, such as tunnel positioning errors or variations in tensioning protocols, both of which are critical determinants of reconstruction success. To minimize surgical variability, all ligament reconstructions in this study were performed by the same experienced surgeon, following strict clinical standards. This included precise tunnel positioning based on anatomical landmarks and controlled graft pretensioning (80 N, 20°–30° knee flexion). Furthermore, previous *in vivo* biomechanical studies have also reported increased knee laxity following ACLR (Zeng et al., 2022; Davis et al., 2019; Slater et al., 2017), suggesting that the procedure may not fully restore native joint stability. Given these findings, we propose that the persistent biomechanical alterations following ACLR are primarily attributable to the surgical procedure itself, rather than inherent limitations of the graft.

The tibial plateau cartilage exhibits regional variability in thickness, collagen alignment, material properties, cellular phenotypes, and tissue organization (Briant et al., 2015; Clark, 1991). Alterations in regional cartilage loading can affect the mechanical strains experienced by embedded chondrocytes, potentially influencing their mechanobiological responses (Briant et al., 2015; Andriacchi et al., 2006). Widespread rise in contact force, coupled with the posterior shift in regional loading, could indicate an over-reliance on the posterior regions of the joint for load-bearing. Such a pattern might signal early markers of cartilage degeneration, osseous changes, or eventual osteoarthritis.

This study highlights the clinical implications of AMD and the limitations of anatomical single-bundle ACLR. Although AMD does not significantly alter peak contact stress, the posterior shift of the CCS and increased medial compartment regional loading suggest that partial injuries can disrupt the joint’s biomechanical environment, potentially predisposing the knee to localized cartilage degeneration and early osteoarthritis. For patients with partial ACL injuries, our findings emphasize three key considerations: 1) surgically optimizing graft thickness to enhance tensile strength and rotational constraints, adjusting tunnel positions to achieve isometric reconstruction for normalized stress distribution, and employing complex techniques like double-bundle or hybrid reconstruction to restore overall knee stability; 2) Rehabilitation should emphasize quadriceps and hamstring strengthening through closed-chain exercises (e.g., single-leg squats) to reduce anterior tibial loading and improve posterior translation, alongside postoperative gait retraining using pressure-sensitive insoles or three-dimensional analysis to normalize AP load patterns; 3) Personalized treatment decisions must integrate patient functional demands and preferences, considering conservative management for low-activity individuals while cautiously weighing the biomechanical trade-offs of surgical interventions in high-functioning patients.

This study has several limitations. First, the study did not employ the more novel technique—selective single-bundle reconstruction (repairing only the damaged anteromedial or posterolateral bundle while preserving the integrity and function of the remaining bundle) (DeFranco and Bach, 2009). Previous research has indicated that forces capable of causing a single-bundle ACL tear often result in substantial interstitial damage to the remaining bundle, potentially lengthening the ligament by more than 50% of its resting length (Noyes et al., 1989). This suggests that selective single-bundle reconstruction may still carry some instability risks. Secondly, the sample size is relatively small, which was constrained by the availability of fresh-frozen cadaveric specimens and the inherent logistical challenges of biomechanical testing. While the sample size aligns with prior cadaveric studies investigating ACL kinematics and contact mechanics, larger cohorts could further enhance the generalizability of our findings. Nevertheless, the repeated-measures design, which utilized each specimen as its own control across conditions, minimized inter-specimen variability and strengthened internal validity. Finally, we only simulated the tension of the quadriceps, without simulating more of the other stable joint structures. Accurately replicating the dynamic synergistic actions of all muscle groups requires sophisticated biomechanical models, such as electromyography-driven multi-muscle control systems. However, the hardware and technology constraints of experimental platform make it challenging to achieve coordinated multi-muscle loading. During the gait cycle, the quadriceps play a crucial role in anterior knee stability. Given that our study specifically investigates the effects of anterior cruciate ligament injury on anteroposterior stability, we prioritized simulating quadriceps tension to isolate the direct consequences of ACL deficiency.

## 5 Conclusion

This study demonstrates that AM bundle injury alters medial compartment loading during gait, causing a slight posterior shift of the center of CCS. And that anatomical single-bundle ACLR does not fully replicate the native ACL’s biomechanical function. Future work should focus on alternative reconstruction techniques and broader injury models to better address these biomechanical challenges.

## Data Availability

The raw data supporting the conclusions of this article will be made available by the authors, upon reasonable request.
